# Historical Hot Spots of Dengue and Zika Viruses to Guide Targeted Vector Control in San Juan, Puerto Rico (2010–2022)

**DOI:** 10.4269/ajtmh.23-0627

**Published:** 2024-02-27

**Authors:** Roberto Barrera, Jose Ruiz, Laura E. Adams, Melissa Marzan-Rodriguez, Gabriela Paz-Bailey

**Affiliations:** ^1^ Dengue Branch, Division of Vector-Borne Diseases, Centers for Disease Control and Prevention, San Juan, Puerto Rico;; ^2^ Department of Health of Puerto Rico, San Juan, Puerto Rico

## Abstract

Dengue viruses (DENV) continue to cause large outbreaks in tropical countries, while chikungunya and Zika (ZIKV) viruses have added complexity to *Aedes*-borne disease prevention and control efforts. Because these viruses are transmitted by the same vectors in urban areas, it is useful to understand if sequential outbreaks caused by these viruses have commonalities, such as similar seasonal and spatial patterns, that would help anticipate and perhaps prevent future outbreaks. We explored and analyzed the heterogeneity of confirmed cases of DENV (2010–2014 and 2015–2022) and ZIKV (2016–2017) during outbreaks in the San Juan metropolitan area of Puerto Rico to explore their degree of overlap and prioritize areas for *Aedes aegypti* control. Deidentified, georeferenced case data were aggregated into grid cells (500 × 500 m) within a geographical information system of the study area and analyzed to calculate the degree of overlap between outbreaks. Spatial autocorrelations using local indicators of spatial associations were conducted to identify significant disease case hot spots and correlations between outbreaks. We found that 75% of cases during the three transmission periods were concentrated in 25% of the total number of grid cells covering the study area. We also found significant clustering of cases during each outbreak, enabling identification of consistent disease hot spots. Our results showed 85% spatial overlap between cases of ZIKV in 2015–2017 and DENV in 2010–2014 and 97% overlap between DENV cases in 2010–2014 and 2015–2022. These results reveal urban areas at greater risk of future arbovirus outbreaks that should be prioritized for vector control.

## INTRODUCTION

Dengue viruses (DENV-1, DENV-2, DENV-3, and DENV-4) are transmitted mainly by the domestic mosquito *Aedes aegypti*, an invasive species with a pantropical distribution.^1^ Other viruses transmitted by this mosquito species, such as chikungunya (CHIKV) and Zika (ZIKV) viruses, have recently expanded their geographic distribution, causing epidemics in areas traditionally affected by dengue viruses.^2^ Although simultaneous circulation of all three arboviruses does not seem to be frequent, there are reports of concurrent circulation of DENV, CHIKV, and ZIKV in the Pacific islands and South America.^3^^–^^5^ Furthermore, a study conducted on the border between Colombia and Venezuela reported coinfections of DENV/CHIKV, DENV/ZIKV, and CHIKV/ZIKV, and 5% of the samples (*n =* 157) were positive for all three arboviruses.^4^

An important question for public health and vector control professionals is how existing data on historical arboviral disease incidence can be used to prevent future outbreaks. Previous studies have shown that the spatial distribution of dengue cases in urban areas is heterogeneous, where cases were concentrated in certain areas or hot spots. A study in Maracay, Venezuela, showed that 70% of all dengue cases were registered in 35% of the urban area. A study in Merida, Mexico, found that 42% of dengue cases occurred in 27% of the city. The authors also reported significant spatiotemporal correlations among cases of dengue, chikungunya, and Zika.^6^ Another study conducted in nine cities in Mexico where dengue was endemic reported overlap between hot spots of dengue, chikungunya, and Zika.^7^ Similarly, hot spots of malaria cases were reported in northern Venezuela, where 9 to 14 of 35 human settlements were thought to be the source of all new infections.^8^

Disease transmission heterogeneity, where a small number of hosts accounts for most of the transmission events, has been studied in a variety of infectious diseases, including vector-borne diseases.^9^ These authors explored the 80:20 rule or pareto rule, where 20% of a host population contributes 80% of the transmission potential.^9^ A study modeling transmission potential indicated that targeted control in hot spots or areas contributing most transmission events should be more efficient than application of homogeneous control.^9^ It has been proposed that controlling dengue transmission in hot spots should reduce transmission in the treated hot spots, as well as in peripheral urban areas, by reducing exports of viruses in infected people or mosquitoes.^10^ Spatial risk stratification for targeted control of *Ae*. *aegypti* may be based on disease incidence, entomological indicators, or vulnerability indices.^7^^,^^10^^,^^11^

Dengue is endemic in Puerto Rico, an unincorporated territory of the United States located in the Caribbean, with outbreaks reported approximately every 3 to 7 years. A large dengue outbreak was reported in 2010, followed by another one in 2012–2013; dengue cases remained at low to endemic levels from 2015 to 2022. DENV-1 was the most prevalent serotype that circulated in both the 2010–2013 and 2015–2022 periods (CDC, unpublished),^12^ potentially limiting the number and extent of cases in an immune population. Zika virus was not reported on the island until 2015 but caused a large outbreak in 2016–2017, with evidence of 20% to 25% of the population infected.^13^ To explore the presence of arboviral hot spots and determine the degree of spatial overlap between outbreaks, we conducted spatial analyses of confirmed cases of DENV in 2010–2014, ZIKV in 2015–2017, and DENV in 2015–2022 in the San Juan metropolitan (metro) area of Puerto Rico. Because these arboviruses are transmitted by the same mosquito vector, we hypothesize that hot spots of DENV and ZIKV cases will show spatial patterns similar to those observed in previous studies.^6^^,^^7^ Detecting the location of arboviral disease hot spots and their spatial-temporal overlap could be used to prioritize areas for disease prevention, such as by conducting *Ae*. *aegypti* control.

## MATERIALS AND METHODS

### Study site.

**Figure 1.**
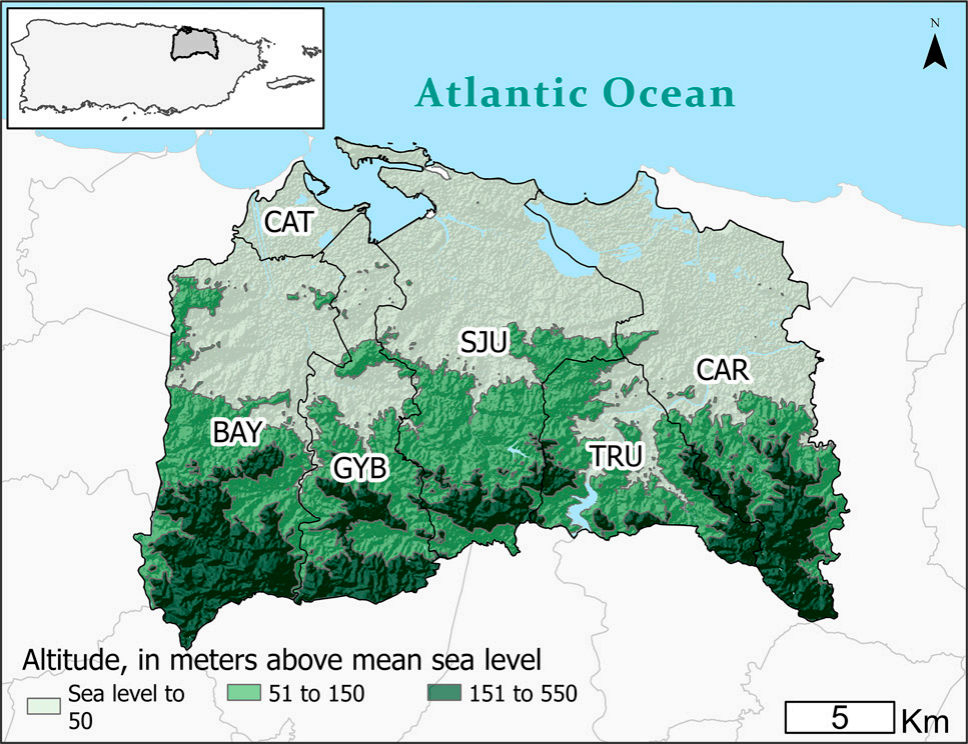
Relief map showing elevation levels (in meters) of the San Juan metropolitan area, Puerto Rico, and limits of municipalities (BAY = Bayamón, CAR = Carolina, CAT = Cataño, GYB = Guaynabo, SJU = San Juan, TRU = Trujillo alto).

The metro area of San Juan, Puerto Rico (506.35 km^2^) is located on the northern Atlantic coast of the main island (Figure 1). It includes the San Juan municipality and five adjacent municipalities (Bayamón, Carolina, Cataño, Guaynabo, and Trujillo Alto). The metro area has a tropical climate, with an average temperature of 27.4°C (average minimum* =* 24.3°C, average maximum = 30.6°C) and mean annual rainfall of 1,784 mm (range, 1,198–2,476 mm) in 2010–2022.^14^ Most communities located along the northern coastal zone of the metro area have elevations between 1 and 550 meters above sea level.

### Arboviral case data collection.

**Figure 2.**
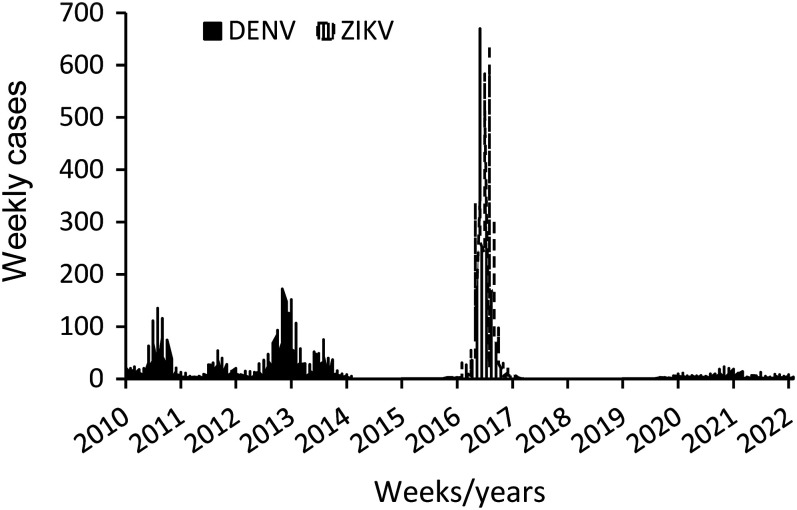
Weekly cases of dengue and Zika from 2010 to 2022 in the San Juan metropolitan area, Puerto Rico.

**Figure 3.**
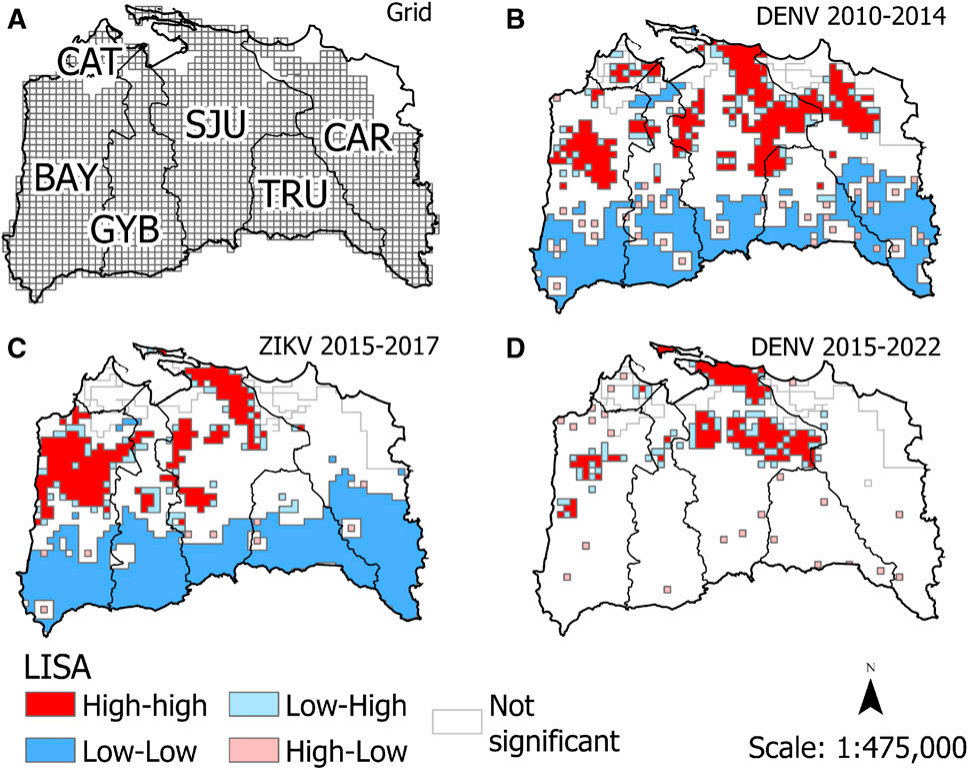
Maps of the San Juan metropolitan area, Puerto Rico, showing the 500 × 500 m grid used to aggregate arboviral disease cases (**A**) and the classification of spatial associations resulting from local indicator of spatial association (LISA) analyses for dengue virus (DENV) cases in 2010–2014 (**B**), Zika virus (ZIKV) cases in 2015–2017 (**C**), and DENV cases in 2015–2022 (**D**).

Arboviral case data came from the Puerto Rico Department of Health arbovirus surveillance system and the Dengue Branch, CDC. Reported human cases of dengue in 2010–2014 were confirmed by reverse transcription-polymerase chain reaction (RT-PCR) and IgM assays, while Zika in 2015–2017 and dengue in 2015–2022 were confirmed by RT-PCR to reduce the possibility of misclassification due to serologic cross-reactivity (Figure 2).^15^^–^^18^ The addresses of cases were geocoded at the household or cross street level to create a layer of location points for geographic information system software’s use and analyses. A grid of 500 × 500 m cells covering the study area (Figure 3A) was created using the Grid Index tool in ArcGIS Pro 2.6 (Environmental Systems Research Institute, Inc., Redlands, CA). The grid was edited to delete grid cells that fell over unpopulated areas (i.e., water bodies, industrial parks, etc.). The edited grid covered 483.90 km^2^ and had 1,937 cells. Cases located within each grid cell were aggregated.

### Statistical analyses.

An indication of the presence of disease hot spots is a large proportion of all cases occurring in a small fraction of the area occupied by a susceptible population. To evaluate this in our dataset, we calculated the percentage of all grid cells that contained 75% of the cases for each transmission period and its average.

To establish if arboviral cases (presence or absence) in grid cells correlated or overlapped between arbovirus outbreaks, we conducted 2 × 2 contingency table analyses (α = 0.05). The null hypothesis is that disease cases’ occurrence between two transmission periods per cell was independent. We calculated three 2 × 2 contingency tables to assess the number of cells with and without any arboviral disease cases reported, comparing the following transmission periods: ZIKV 2015–2017 to DENV 2010–2014, ZIKV 2015–2017 to DENV 2015–2022, and DENV 2010–2014 to DENV 2015–2022. The strength of the association for each comparison was measured using Cramer’s V, which varies from 0 (lack of association) to 1 (perfect association; α = 0.05). We also calculated the odds ratios of cases of one arbovirus transmission period (e.g., DENV in 2015–2022) occurring in cells where another arbovirus transmission period occurred (e.g., DENV in 2010–2014) in the city. The odds ratio provides another indication of the strength of the association between pairs of transmission periods.

We determined if there were global spatial autocorrelations or clustering of cases for each of the three arbovirus outbreaks using Moran’s I (α = 0.05). Provided that the Moran’s I was significant, we also investigated the presence and significance of hot spots in each of the three time periods by using the univariate local indicator of spatial association (LISA),^19^ which reveals the degree of significant (α = 0.05) spatial clustering around each cell. A hot spot was defined as a cell with above average case numbers surrounded by cells with above average case numbers. A cold spot was defined as a cell with low case numbers surrounded by cells with low case numbers. Outlier cells were defined as cells with high case numbers surrounded by cells with low case numbers, or vice versa (low and high).^19^ Hot spot analyses for the three arbovirus transmission periods were performed in GeoDa 1.20.^20^ A contiguity-based spatial weighted matrix was created using the grid and the queen criterion to calculate the neighbor relationships between cells. The queen criterion defines a neighbor cell if it shares a common edge or vertex.

Bivariate LISA (BiLISA), an extension of the univariate LISA analysis, was performed to assess the local spatial correlations between one transmission period and a previous or future one. In the BiLISA, case numbers of a given cell in one transmission period (e.g., DENV 2015–2022) is compared or plotted against the weighted average of cases in corresponding neighboring cells from another transmission period, also called spatial lags (e.g., DENV 2010–2014). This analysis indicates if a cluster of cases during one transmission period occurred at similar locations as those in another transmission period. We conducted BiLISA analyses (α = 0.05) for the three pairs of transmission periods. As part of the BiLISA analyses, we report on the results of least square regressions that simultaneously evaluate both the direct correlation between cases of two transmission periods (e.g., cell-to-cell correlations between DENV 2010–2014 and ZIKV 2015–2017) and the correlation between the spatial lags or neighboring cell values of one arbovirus transmission period and the other (e.g., neighboring cells from DENV 2010–2014 around a center cell from ZIKV 2015–2017). These regression analyses are useful for understanding how cases in two transmission periods were spatially correlated while controlling for the direct correlations to avoid overestimating the spatial component of the correlation. Another way to interpret these results is how predictive the occurrence of case clustering of a future transmission period is explained by data coming from a previous transmission period, assuming that the transmission dynamics of the same or different viruses being compared in both periods were similar. These analyses were conducted using GeoDa 1.20.^21^

## RESULTS

A total of 7,918 dengue cases from January 2010 to February 2014, 9,963 Zika cases from December 2015 to June 2017, and 819 dengue cases from January 2015 to February 2022 were reported and had geolocations available. We found that most cases during the three time periods (75%; 14,032) were concentrated in 24.5% of the total number of 500 × 500 m cells covering the study area (Table 1). The transmission period of DENV in 2015–2022 had 75% of cases concentrated in fewer cells (10.4%), whereas a larger percentage of cells concentrated 75% of cases during the other transmission periods (21.1–23.4%) (Table 1).

**Table 1:** Percentage of grid cells covering the study area that contained 75% of cases in each epidemic in the San Juan metropolitan area, Puerto Rico

Outbreaks	Total Cases	75% of Cases	% of Grid Cells with 75% of Cases
DENV 2010–2014	7,918	5,941	23.4
ZIKV 2015–2017	9,963	7,472	21.1
DENV 2015–2022	819	614	10.4
Total	18,700	14,032	24.5

DENV = dengue virus; ZIKV = Zika virus.

The association between grid cells with and without cases of Zika in 2015–2017 and dengue in 2010–2014 across the two transmission periods was significant (χ^2^ = 797.1; *P* <0.001), with a significant positive association (Cramer’s V = 0.64) between case occurrences in the San Juan metro area (Table 2). The odds of cells with Zika in 2015–2017 was 21.3 times higher in cells with previous DENV in 2010–2014 than in cells without DENV. This result showed that 85% of Zika cases in 2015–2017 occurred in grid cells that had dengue cases in 2010–2014. The Spearman correlation (*r_s_*) analysis between cases of Zika in 2015–2017 and dengue in 2010–2014 was positive and significant (*r_s_* = 0.76 [*P <*0.001; *N =* 1,937]).

**Table 2:** Number (percentage) of grid cells with and without cases of DENV in 2010–2014 and ZIKV in 2015–2017 in the San Juan metropolitan area, Puerto Rico

DENV 2010–2014	ZIKV 2015–2017		
	Cases	No Cases	Row Totals
Cases	917 (83.7)	178 (16.3)	1,095 (56.5)
No cases	164 (19.5)	678 (80.5)	842 (43.5)
Column totals	1,081 (55.8)	856 (44.2)	1,937

DENV = dengue virus; ZIKV = Zika virus.

Analysis of association of case occurrence between DENV 2010–2014 and DENV 2015–2022 transmission periods was significant (χ^2^ = 343.1; *P <*0.001), with a positive association (Cramer’s V = 0.42, *P <*0.001) and an odds ratio of 38.9, indicating a high occurrence of dengue cases in 2015–2022 in places with dengue cases in 2010–2014 (Table 3). This result showed that 97% of dengue cases in 2015–2022 occurred in cells where dengue cases were previously reported in 2010–2014. The Spearman correlation analysis between cases of DENV in 2010–2014 and DENV in 2015–2022 was positive and significant (*r_s_* = 0.54 [*P <*0.001; *N =* 1,937]). The analysis of cooccurrence between dengue cases in 2015–2022 and Zika cases in 2015–2017 was also significant (χ^2^ = 345.8; *P <*0.001), with a positive association (Cramer’s V = 0.42, *P <*0.001) and an odds ratio of 34.2 (Table 4). This result showed that 96.5% of dengue cases in 2015–2022 occurred in cells with ZIKV in 2015–2017. The Spearman correlation analysis between cases of ZIKV in 2016–2017 and DENV in 2015–2022 was positive and significant (*r_s_* = 0.51 [*P <*0.001; *N =* 1,937]).

**Table 3:** Number (percentage) of grid cells with and without cases of DENV in 2015–2022 and DENV in 2010–2014 in the San Juan metropolitan area, Puerto Rico

DENV 2010–2014	DENV 2015–2022		
	Cases	No Cases	Row Totals
Cases	394 (36)	70 (64)	1,095 (56.5)
No cases	12 (1.4)	830 (98.6)	842 (43.5)
Column totals	406 (21)	1,531 (79)	1,937

DENV = dengue virus.

**Table 4:** Number (percentage) of grid cells with and without cases of DENV in 2015–2022 and ZIKV in 2015–2017 in the San Juan metropolitan area, Puerto Rico

ZIKV 2015–2017	DENV 2015–2022		
	Cases	No Cases	Row Totals
Cases	392 (36.3)	689 (63.7)	1,081 (55.8)
No cases	14 (1.6)	842 (98.4)	856 (44.2)
Column totals	406 (21)	15,31 (79)	1,937

DENV = dengue virus; ZIKV = Zika virus.

Global Moran’s I values for the DENV 2010–2014 (0.43), ZIKV 2015–2017 (0.40), and DENV 2015–2022 (0.32) transmission periods were significant (*P* <0.001), showing positive global spatial autocorrelations. The univariate LISA analyses showed that hot spots of cases during the DENV 2010–2014 transmission period were present in all six metro municipalities, with greater frequency in the San Juan, Carolina, and Bayamón municipalities (Figure 3B), whereas most hot spots during the DENV 2015–2022 transmission period were concentrated in the San Juan municipality (Figure 3D). Hot spots during the ZIKV 2015–2017 period were mostly concentrated in the San Juan and Bayamón municipalities (Figure 3C). Cold spots were most frequently detected in the southern regions of the municipalities, where the population is scattered over mountainous areas; this occurred during both the DENV 2010–2014 and ZIKV 2015–2017 transmission periods. The southern regions also had numerous small areas with high number of cases neighboring areas with low number of cases during all three transmission periods, showing arbovirus transmission in isolated communities surrounded by uninhabited or scarcely inhabited areas.

**Figure 4.**
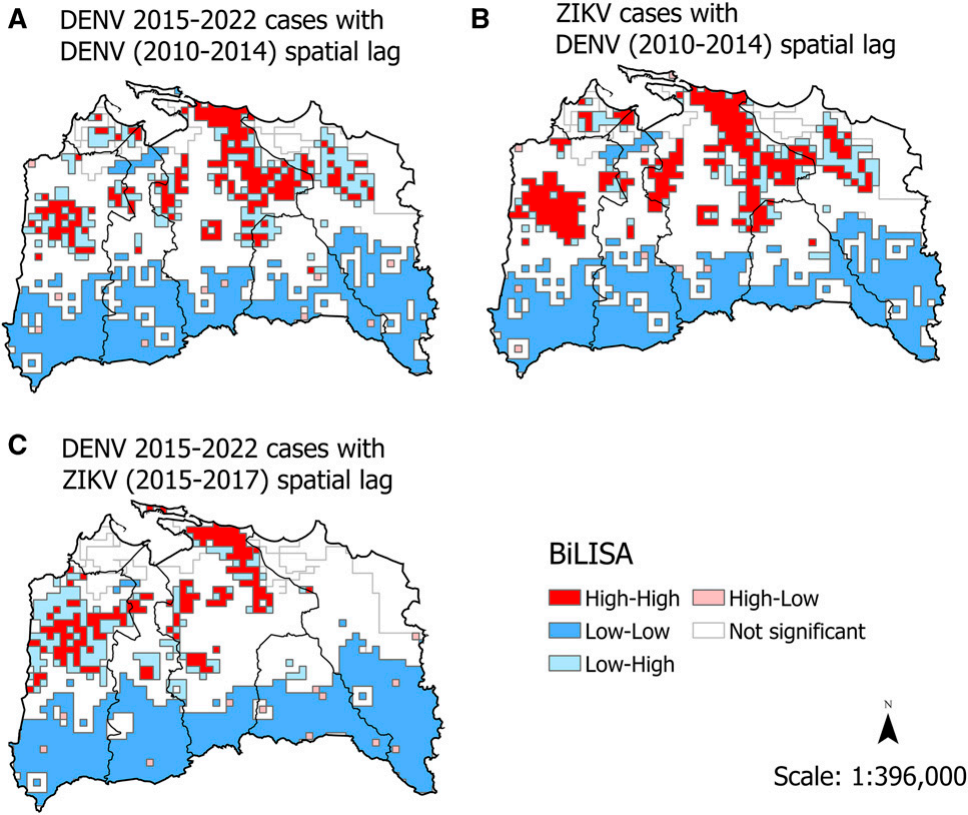
Bivariate local indicator of spatial association (BiLISA) analyses of spatial associations between transmission periods of dengue virus (DENV) in 2015–2022 and DENV in 2010–2014 (**A**), Zika virus (ZIKV) in 2015–2017 and DENV in 2010–2014 (**B**), and DENV in 2015–2022 and ZIKV in 2015–2017 (**C**) in the San Juan metropolitan area, Puerto Rico.

We used bivariate LISA analyses to examine how cases during a given transmission period were spatially correlated with previous transmission trends to see how well the spatial pattern of cases during a previous arbovirus outbreak was predictive of the spatial pattern of a subsequent outbreak (e.g., significant positive spatial correlations). The analyses correlating arbovirus cases during the DENV 2015–2022 transmission period with cases reported during the previous DENV 2010–2014 period (Figure 4A) were significant for direct correlations (cells with any cases in DENV 2010–2014) and indirect correlations (in comparison to dengue case counts in neighboring cells in 2010–2014) (Supplemental Table 1). The pattern of DENV hot spots in 2015–2022 (Figure 3D) compared with the previous DENV 2010–2014 transmission period (Figure 4A) indicated substantial spatial overlap, although the main circulating dengue virus in both outbreaks was DENV-1. The bivariate LISA analyses (Figure 4B) of the correlations between ZIKV 2015–2017 and the previous DENV 2010–2014 transmission period were significant for both direct and indirect correlations (Supplemental Table 2). There was substantial overlap of Zika cases in areas with high dengue case numbers in 2010–2014 (Figure 4B). Lastly, the bivariate analysis of dengue cases in 2015–2022 with the previous ZIKV outbreak in 2015–2017 was also highly significant for direct and indirect correlations (Supplemental Table 3). Again, the spatial pattern of dengue cases in 2015–2022 closely resembled the case distribution patterns seen during ZIKV transmission in 2015–2017 (Figure 4C).

## DISCUSSION

We investigated the spatial patterns of arboviral cases during sequential transmission periods of DENV (2010–2014 and 2015–2022) and ZIKV (2015-2017) in the San Juan metro area to determine if there were hot spots of arboviral transmission and whether these overlapped in subsequent transmission periods. The analyses of global and local indicators of spatial association for each transmission period were significant, enabling the identification of hot spots during each period. These results concur with previous studies showing clustering of dengue cases in hot spots during epidemics.^22^^–^^24^ Less information was available about whether epidemics of dengue and other arboviruses transmitted by *Ae*. *aegypti* show similar spatial clustering patterns. Our results contribute to the literature by showing that Zika cases in 2015–2017 occurred in 85% of grid cells where dengue cases were reported in 2010–2014. Overlap of dengue cases in 2015–2022 with cases during 2010–2014 was even higher, with 97% overlap. It is interesting to note that the dengue cases in 2010–2014 were caused mainly by DENV-1 and DENV-4 and most cases during 2015–2022 were also caused by DENV-1, indicating that the pool of people susceptible to this serotype was not exhausted during the previous large epidemics. The large overlap observed between both DENV and ZIKV epidemics is greater than that observed in a previous study conducted in Merida, Mexico, where the authors reported significant spatiotemporal correspondence among DENV, CHIKV, and ZIKV epidemics.^6^^,^^7^ Thus, analyses of historical data on arboviruses transmitted by *Ae*. *aegypti* are useful to anticipate locations within the city where eventual epidemics of arboviruses transmitted by this mosquito will concentrate.

We were also interested in examining if arbovirus cases in the study areas conformed to the 80:20 pareto rule,^9^ whereby most arbovirus cases are reported in people occupying a relatively small portion of the urban area. In this study, most cases during all three transmission periods (75%) were concentrated in a smaller area of the city (25%). Identifying areas with high and recurrent arbovirus cases could help allocate disease control resources to these priority areas with the hope of preventing future epidemics. How well these results will hold during future epidemics of arboviruses transmitted by *Ae*. *aegypti* in the San Juan metro area is unknown. However, the high overlap observed between ZIKV transmission and a previous DENV epidemic is reassuring, as there was great emphasis at detecting and confirming ZIKV cases during the 2015–2017 epidemic in Puerto Rico,^25^ given the risk of severe infection outcomes, such as Guillain-Barré syndrome and microcephaly.^26^ Areas identified as high risk based on past occurrence of cases may change over time, so maps should be frequently updated, especially if effective vector control was conducted.

Studying spatial and temporal patterns of DENV and ZIKV using data generated by passive surveillance systems is limited by incomplete case capture, as many people with DENV and ZIKV infections are asymptomatic, have mild symptoms, do not consult a physician, or do not get tested.^27^ Another important limitation of the study is that the location of confirmed cases was reported as the domicile of the patient, although not all infections may have happened at home; additionally, georeferencing is not available for all addresses in Puerto Rico and may have been more or less available for some parts of the San Juan metro area (e.g., likely less reliable for rural areas).

In conclusion, the concentration of a large number of arbovirus cases in a smaller portion of the study area, the presence of hot spots, and the significant spatial overlap of cases in the three transmission periods investigated could be used to define priority areas for preventive *Ae*. *aegypti* control, particularly those under resource-limited conditions. Preventive *Ae*. *aegypti* control implies keeping this mosquito below a critical density threshold that prevents or reduces local outbreaks,^28^ at least during interepidemic periods, to prevent epidemics from building up later.^29^^,^^30^

## Supplemental Materials

10.4269/ajtmh.23-0627 Supplemental Materials
