# Supplementary material for: Historical Hot Spots of Dengue and Zika Viruses to Guide Targeted Vector Control in San Juan, Puerto Rico (2010–2022)
Source: Am J Trop Med Hyg. 2024 Feb 27;110(4):731–7. doi: 10.4269/ajtmh.23-0627 (PMC10993837; doi:10.4269/ajtmh.23-0627)
Supplement: Supplemental Materials [file tpmd230627.SD1.pdf]

Table S1: Multiple regression analysis of cases of DENV during 2015-2022 with previous cases of DENV 2010-2014 and with the spatial lags of DENV 2010-2013 in the Metro Area of San Juan, Puerto Rico.

Variable	Coeff.	Std. error	t-Statistic	Probability
Constant	-0.0048	0.0199	-0.2401	0.8103
DENV 2010-2014	0.3353	0.0251	13.3747	0.0000
DENV 2010-2014 spatial lag	0.2922	0.0360	8.1212	0.0000

Adjusted R-squared: 0.236487

Table S2: Multiple regression analysis of cases of ZIKV in 2015-2017 with previous cases of DENV 2010-2014 and with the spatial lags of DENV 2010-2014 in the Metro Area of San Juan, Puerto Rico.

Variable	Coeff.	Std. error	t-Statistic	Probability
Constant	-0.0052	0.0185	-0.2800	0.7795
DENV 2010-2014	0.4230	0.0233	18.1569	0.0000
DENV 2010-2014 spatial lag	0.3167	0.0334	9.4733	0.0000

Adjusted R-squared: 0.340889

Table S3: Multiple regression analysis of cases of DENV during 2015-2022 with previous cases of ZIKV 2015-2017 and with the spatial lags of ZIKV 2015-2017 in the Metro Area of San Juan, Puerto Rico.

Variable	Coeff.	Std. error	t-Statistic	Probability
Constant	-0.0035	0.0209	-0.1691	0.8658
ZIKV 2015-2017	0.1947	0.0258	7.5379	0.0000
ZIKV 2015-2017 spatial lag	0.3715	0.0383	9.6953	0.0000

Adjusted R-squared: 0.157155
